# Direct characterization of the native structure and mechanics of cyanobacterial carboxysomes†
†Electronic supplementary information (ESI) available: 7 figures, 2 tables and 1 file. See DOI: 10.1039/c7nr02524f


**DOI:** 10.1039/c7nr02524f

**Published:** 2017-06-08

**Authors:** Matthew Faulkner, Jorge Rodriguez-Ramos, Gregory F. Dykes, Siân V. Owen, Selene Casella, Deborah M. Simpson, Robert J. Beynon, Lu-Ning Liu

**Affiliations:** a Institute of Integrative Biology , University of Liverpool , Liverpool L69 7ZB , UK . Email: luning.liu@liverpool.ac.uk

## Abstract

Carboxysomes are proteinaceous organelles that play essential roles in enhancing carbon fixation in cyanobacteria and some proteobacteria. These self-assembling organelles encapsulate Ribulose 1,5-bisphosphate carboxylase/oxygenase (Rubisco) and carbonic anhydrase using a protein shell structurally resembling an icosahedral viral capsid. The protein shell serves as a physical barrier to protect enzymes from the cytosol and a selectively permeable membrane to mediate transport of enzyme substrates and products. The structural and mechanical nature of native carboxysomes remain unclear. Here, we isolate functional β-carboxysomes from the cyanobacterium *Synechococcus elongatus* PCC7942 and perform the first characterization of the macromolecular architecture and inherent physical mechanics of single β-carboxysomes using electron microscopy, atomic force microscopy (AFM) and proteomics. Our results illustrate that the intact β-carboxysome comprises three structural domains, a single-layered icosahedral shell, an inner layer and paracrystalline arrays of interior Rubisco. We also observe the protein organization of the shell and partial β-carboxysomes that likely serve as the β-carboxysome assembly intermediates. Furthermore, the topography and intrinsic mechanics of functional β-carboxysomes are determined in native conditions using AFM and AFM-based nanoindentation, revealing the flexible organization and soft mechanical properties of β-carboxysomes compared to rigid viruses. Our study provides new insights into the natural characteristics of β-carboxysome organization and nanomechanics, which can be extended to diverse bacterial microcompartments and are important considerations for the design and engineering of functional carboxysomes in other organisms to supercharge photosynthesis. It offers an approach for inspecting the structural and mechanical features of synthetic metabolic organelles and protein scaffolds in bioengineering.

## Abstract 2

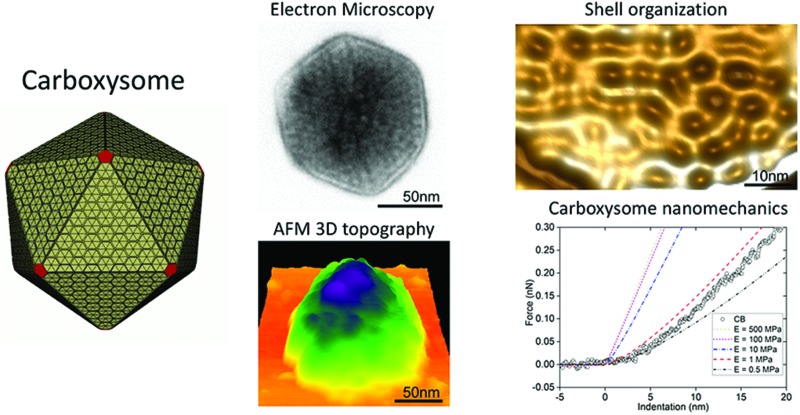
The spatial protein organization, topography and physical mechanics of native carboxysomes, the self-assembling carbon-fixation machinery in cyanobacteria, were characterized.

## Introduction

Compartmentalization of metabolic pathways in cells is key for enhancing and modulating cellular metabolism in space and time.^1^,^2^ Particularly versatile paradigms in prokaryotes are bacterial microcompartments (BMCs) that are widespread among bacterial phyla.^3^ They sequester diverse enzymes that catalyze sequential metabolic reactions from the cytosol and play important roles in CO_2_ fixation, pathogenesis, and microbial ecology.^4^–^6^ While the full inventory of the metabolic diversity of BMCs is still being uncovered, the common architectural features of all BMCs are that they are made entirely of protein and comprise an outer icosahedral shell and encased interior enzymes. The protein shell, structurally resembling virus capsids, is made of multiple protein paralogs forming hexagons and pentagons, and acts as a physical barrier that controls the passage of substrates and products of enzymatic reactions.

The carboxysome is one such BMC found in cyanobacteria and some chemoautotrophs.^7^,^8^ Carboxysomes carry out the final stages of the CO_2_-concentrating mechanism of cyanobacteria and play a central role in the Calvin-Benson-Bassham cycle, and thus provide impacts on photosynthetic carbon fixation and global primary production.^9^ These organelles encapsulate the CO_2_-fixing enzymes, Ribulose 1,5-bisphosphate carboxylase/oxygenase (Rubisco) and β-carbonic anhydrases (β-CA) within a selectively permeable shell that allows for the diffusion of HCO_3_^–^ and prevents CO_2_ from leaking into the cytosol.^10^ Based on the types of Rubisco enzymes, gene organization and protein composition, carboxysomes can be divided into two categories, α-carboxysomes that possess Form 1A Rubisco and β-carboxysomes that sequester plant-like Form 1B Rubisco.^11^–^13^ The colocalized β-CA convert HCO_3_^–^ to CO_2_ and create a CO_2_-rich environment in the carboxysomal lumen to favor the carboxylase activity of Rubisco. As a consequence, this highly-organized structure results in high levels of CO_2_ in the vicinity of Rubisco, thereby enhancing carbon fixation. Given their self-assembly, modularity and encapsulation attributes, there is a growing interest in constructing carboxysomes into other organisms using synthetic biology, with the intent of supercharging photosynthesis and developing new bio-nanoreactors and protein scaffolds for metabolic enhancement and molecule delivery.^14^–^16^ However, the inherent properties of the organization and mechanics of functional carboxysomes await in-depth experimental investigation.

The model cyanobacterium *Synechococcus elongatus* PCC7942 (Syn7942) contains β-carboxysomes. The shell of β-carboxysomes from Syn7942 is composed of the structural proteins CcmK2, CcmK3 and CcmK4, which appear as hexamers and form the shell facets,^17^ the CcmL pentamers that sit at the vertices between the shell facets,^18^ as well as CcmO that is deduced to interface the edges of shell facets.^19^ The core of β-carboxysomes is formed by Form 1B Rubisco, the β-CA (CcaA), CcmM and CcmN.^20^ CcmM has two active isoforms, CcmM58 and CcmM35, with distinct functions.^21^ CcmM58 provides the interactions between the outer shell and β-CA and Rubisco molecules adjacent to the shell; whereas the 35 kDa truncated version CcmM35 is likely located in the carboxysomal lumen and crosslinks Rubisco enzymes.^22^,^23^ CcmN acts as a bridge between CcmM and the shell by its two functional domains. The N-terminal domain of CcmN interacts with CcmM58 and the C-terminal peptide is capable of binding the major shell protein CcmK2.^24^ In addition, the β-carboxysome shell also contains the minor protein CcmP that forms a dimer of trimers and likely modulates the shell permeability.^25^ RbcX is recognized as a chaperonin-like protein for Rubisco assembly, but its precise function in Syn7942 is still unclear.^26^,^27^ To date, models of the β-carboxysome are based on crystal structures of individual β-carboxysome proteins with the assumption of icosahedral symmetry.^18^ The molecular details of the β-carboxysome structure remains unclear.

Three distinct assembly pathways of carboxysome modules have been deduced. In Syn7942, *de novo* assembly of β-carboxysomes exploits the “inside out” mode, Rubisco and CcmM first forming the core, followed by the encapsulation of shell proteins.^28^,^29^ In contrast, the formation of empty α-carboxysome shells in a Rubisco-knockout mutant of the chemoautotroph *Halothiobacillus neapolitanus* led to the implicit assumption that the shell forms first during α-carboxysome biogenesis.^30^,^31^ In addition, partial α-carboxysomes composed of the fractional shell and attached layers of Rubisco enzymes were imaged in *H. neapolitanus* and no Rubisco aggregations were observed,^32^ suggesting a simultaneous assembly pathway for carboxysome biogenesis.

Within the cytosol which is a crowded and changing environment,^33^ it is important that carboxysomes are sufficiently robust to ensure the proper protein assembly, encapsulation of Rubisco enzymes and functional architecture. On the other hand, they are also flexible and dynamic to allow metabolite passage, turnover of building modules and interactions with other cellular components. Indeed, protein modules in the BMC shell facet are highly dynamic.^34^ Through specific interactions with the cytoskeleton, β-carboxysomes in Syn7942 are evenly positioned along the longitudinal axis of the cell, ensuring equal segregation of these essential organelles to daughter cells.^35^ The biosynthesis and spatial organization of β-carboxysomes in Syn7942 also have a close correlation with photosynthetic electron flow regulated by light.^36^ In such a dynamic context, the inherent physical properties of carboxysomes are important for the structural and functional integrity and flexibility of the icosahedral organelles. Until now, the exact mechanical nature of carboxysomes has not been characterized.

In this work, we purified functional β-carboxysomes from Syn7942 and carried out the first detailed characterization of the three-dimensional structure, topography and intrinsic nanomechanics of native β-carboxysomes using transmission electron microscopy (TEM), atomic force microscopy (AFM) and proteomics. Our results reveal three distinct structural domains of intact β-carboxysomes, the native protein organization of the shell and the specific protein interactions in partial carboxysomes. Though structurally resembling virus capsids, β-carboxysomes present significantly soft mechanics. The study provides novel insights into the inherent structure and physical elasticity of native β-carboxysomes. It will empower our toolbox for the design and construction of functional metabolic machinery with applications in bioengineering and nanotechnology.

## Results and discussion

### Isolation of functional β-carboxysomes from Syn7942

**Fig. 1:**
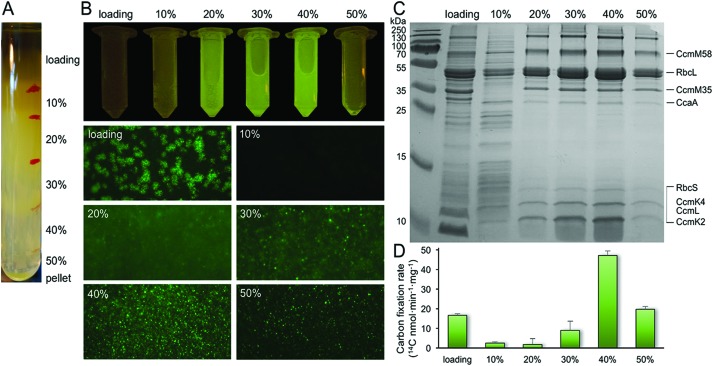
Isolation and characterization of CcmK4:eGFP β-carboxysomes from Syn7942. (A) Step sucrose gradient separation of CcmK4:eGFP β-carboxysomes. (B) Fluorescence detection of β-carboxysomes fused with GFP in different sucrose fractions. (C) SDS-PAGE of individual fractions from the β-carboxysome purification, showing the polypeptide composition of isolated β-carboxysomes. The presence of Rubisco was verified by immunoblot analysis (Fig. S1†). Determination of β-carboxysome proteins was confirmed by proteomic analysis (Table S1†). (D) Rubisco activities of each sucrose fractions determined by ^14^C radiometric assay.

Purification of α-carboxysomes has led to the extensive characterization of α-carboxysome structure.^32^,^37^–^39^ In contrast, no successful isolation of functional β-carboxysomes has yet been developed,^12^ hampering the study of β-carboxysome structure. Here, we use a CcmK4:eGFP Syn7942 strain to develop the procedure for β-carboxysome purification. The GFP tagging, with undetectable effects on the β-carboxysome structure and physiology,^36^ enables us to fluorescently screen the β-carboxysome fractionation during the isolation and characterization processes. Syn7942 cells were grown under high light (∼100 μE m^–2^ s^–1^) to increase the carboxysome abundance per cell, according to the previous study.^36^ Following Triton X-100 treatment, β-carboxysomes were enriched in the pellet by two steps of centrifugation and many cellular components remained in the supernatant. After sucrose gradient centrifugation (Fig. 1A), most of the rest cellular components appeared in the top and pellet of sucrose gradient fractions by proteomics and TEM (data not shown). The majority of β-carboxysomes were determined in the 20%, 30% and 40% fractions by fluorescence imaging (Fig. 1A). Most of the strong GFP spots appear in the 40% sucrose gradient fraction (Fig. 1B). SDS-PAGE illustrates the polypeptide patterns of β-carboxysomes in each fraction (Fig. 1C). Rubisco enzymes are the most abundant components in all fractions, in agreement with immunoblot data (Fig. S1†). Carbon fixation assays of each β-carboxysome fractions reveal that the 40% fraction presents the highest Rubisco activity. Proteomic analysis of the 40% fraction allows the identification of a total of seven β-carboxysome components, including the shell proteins (CcmK2, CcmK4, CcmL), shell-associated proteins (CcmM, CcaA) and internal proteins (RbcL, RbcS) (Table S1, ESI File 1†). These results verify the proper fractionation of functional β-carboxysomes from Syn7942 (40%), whereas the 20 and 30% fractions may contain β-carboxysome subcomplexes.

In addition to Rubisco molecules, CcmM are relatively abundant in the β-carboxysome, in line with their deduced roles in interlinking Rubisco enzymes to form the paracrystalline arrays and interacting with the shell.^13^ We could not differentiate the two CcmM isoforms, CcmM58 and CcmM35, in the isolated β-carboxysomes, due to the absence of specific peptide sequences in the N-terminus of CcmM58 identifiable in mass spectroscopy. The minor shell protein CcmL was identifiable in the isolated β-carboxysomes. According to the icosahedral shape, twelve CcmL pentamers are required per carboxysome. Surprisingly, although CcmO was deduced to occupy 10–30% of the shell surface,^19^ it was not detectable in the isolated β-carboxysomes by mass spectroscopy. Likewise, CcmN, CcmP and RbcX were not detected neither in this work nor in the previous study,^40^ indicative of their low abundance in the β-carboxysome (compared to CcmL), the weak interactions with other carboxysome proteins, or changeable carboxysome composition in different conditions. Further exploration is needed to examine the accurate stoichiometry and function of these undetectable components in β-carboxysomes.

Apart from the predominant β-carboxysome components, four cytoskeletal proteins (ParA, MreB, FtsZ, Ftn2) were identified in relatively high abundances in the 40% fraction (ESI File 1†), supporting the notion that there are inherent interactions between β-carboxysomes and the cytoskeleton, which is key to the spatial positioning of β-carboxysomes in Syn7942.^35^ It is feasible that the GFP tags of CcmK4 somehow eliminate potential associations between β-carboxysomes and other cellular structures, *albeit* the underlying mechanism remains unclear.

### Structures of β-carboxysome fragments and intact β-carboxysomes

**Fig. 2:**
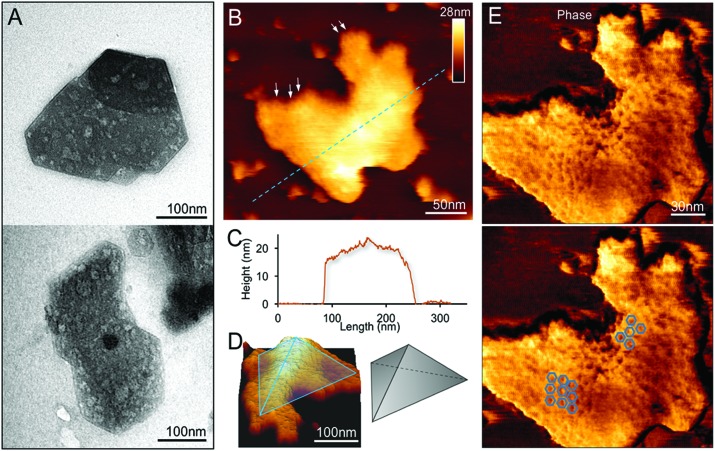
Characterization of the β-carboxysome fragments in the 20% and 30% fractions. (A) TEM images of β-carboxysome fragments captured in the 20 and 30% fractions. Regular and straight facet edges and proteins associated with the facets were observed. More TEM images are shown in Fig. S2.† (B) AFM topograph of a typical β-carboxysome fragment illustrating the spatial organization of individual shell proteins (indicated by arrows). AFM topograph of a curved β-carboxysome fragment is shown in Fig. 3. (C) Cross-section analysis of the β-carboxysome fragment along the dashed line indicated in (B). (D) 3D representation of the β-carboxysome fragment, showing the possible shell substructure comprises two shell facets that have a joint facet edge. (E) AFM phase image recorded together with the height image (B), displaying the native protein organization in the shell facets, with patterns of individual shell hexamers highlighted in blue hexagons.

We examined the structures of isolated β-carboxysomes using TEM and AFM. Electron micrographs of negatively stained specimens demonstrate that the 20 and 30% sucrose gradient fractions contain predominantly the β-carboxysome substructures (Fig. 2A and Fig. S2†). Shell facets with straight and regular edges as well as proteins attached to the shell were visualized. AFM imaging in solution was used to characterize the native topography of β-carboxysome subcomplexes at near physiological conditions (Fig. 2B–E). Cross-section analysis reveals that the thickness of these carboxysome fragments is 18.03 ± 8.11 nm (*n* = 20), with a range from 12.1 to 25.3 nm (Fig. 2C). They are thicker than a single shell protein layer that is about 4.0 nm thick.^17^,^34^ Three-dimensional AFM image and cross-section analysis suggest that the β-carboxysome fragments observed is composed of two shell facets with a joint edge that is raised from the AFM substrate surface (Fig. 2C and D). Individual shell hexamers and their spatial organization in the shell facets could be seen (Fig. 2E).

**Fig. 3:**
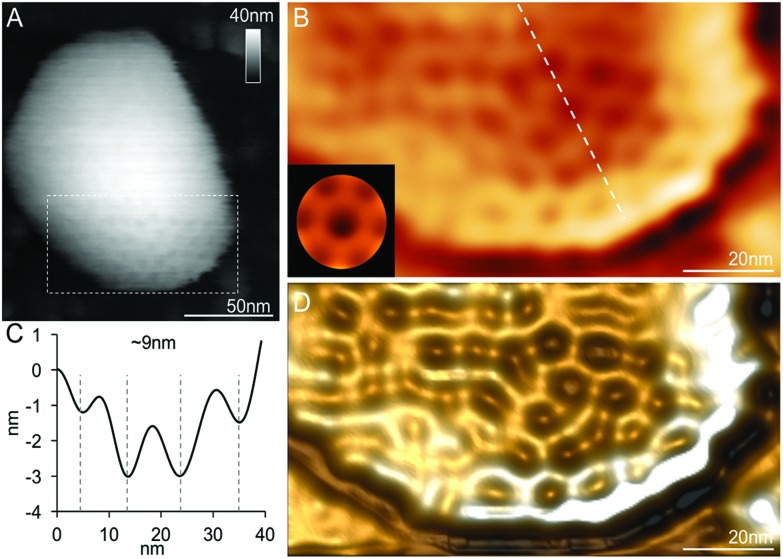
Spatial organization of proteins in a partial β-carboxysome from the 30% fraction. (A) High-resolution AFM topograph of a partial β-carboxysome fragment in buffer. (B) High-pass-filtered AFM image showing the protein organization in the shell fragment depicted in (A). The white line of the cross section was used to calculate the pattern of hexamer organization in (C). (C) The cross-section profile illustrates the periodic arrangement of hexamers and the center-to-center distance between neighboring hexamers is ∼9 nm. (D) Three-dimensional height image of the shell showing the shell protein structures and arrangement.

In addition to the relatively flat shell sheets, more curved shell fragments were also imaged in solution. Fig. 3A and B show one curved shell patch where the native large-scale organization of shell hexamers can be viewed, reminiscent of the organization of shell hexamers in synthetic BMC shell self-assemblies observed using AFM.^34^ Cross-section analysis reveals the periodic arrangement of shell hexamers and their center-to-center distance is ∼9 nm (Fig. 3B and C). The protein structures and arrangement in the shell were better discerned in the 3D height AFM image (Fig. 3D).

Our EM and AFM results, together with the SDS-PAGE (Fig. 1C), Rubisco assay (Fig. 1D) and immunoblot analysis (Fig. S1†), reveal that the observed specimens are partial β-carboxysome modules (12.1–25.3 nm thick) that comprise shell facets, plus shell-associated proteins and 1–2 layers of Rubisco enzymes. Despite the possible artifacts in sample purification, these β-carboxysome substructures resemble the partial α-carboxysomes observed previously,^32^ probably acting as intermediates generated in the β-carboxysome biogenesis or degradation pathways.

**Fig. 4:**
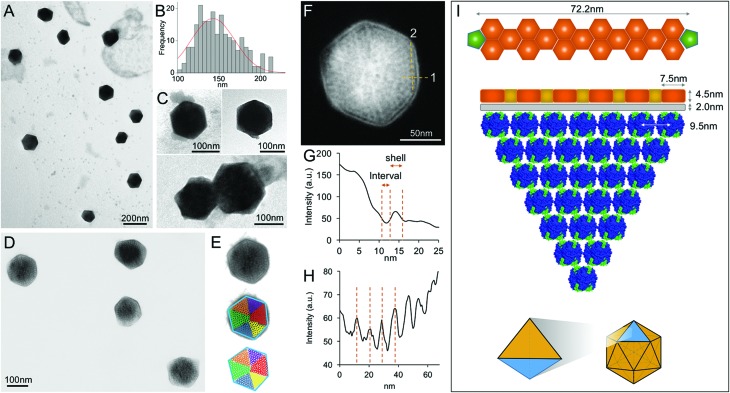
Characterization of the intact β-carboxysomes in the 40% fractions. (A) An overview TEM image of the 40% sucrose fraction showing individual β-carboxysomes with the polyhedral shape. (B) Histogram of the diameters of β-carboxysomes measured from TEM images shows the size heterogeneity of β-carboxysomes (*n* = 90). Each measurement is the mean of the three vertex-to-vertex measurements from a single carboxysome as described in Fig. S3.† (C) Typical TEM images of individual intact β-carboxysomes (top) and β-carboxysome aggregations (bottom). More TEM images are shown in Fig. S3.† (D) High-resolution TEM imaging allows the direct visualization of both β-carboxysome shell and internal structures. (E) Zoomed-in view of a single β-carboxysome with the resolved structural features highlighted. The outer shell is highlighted by a blue hexagon, and the Rubisco molecules are highlighted by circles color coded by individual interior Rubisco-organizing pyramids. (F) Measurement of the shell thickness, inner gap (line 1) and Rubisco packing (line 2). (G) Profile analysis along the line 1 in (F), indicating the shell thickness of 4.5 nm and the inner gap (2.0 nm) between the shell and Rubisco-organizing structure. (H) Profile analysis along the line 2 in (F), indicating the periodicity of Rubisco arrangement (∼9.5 nm). (I) A structural model of the β-carboxysome from Syn7942, based on the TEM observations. Top, the model of β-carboxysome facet edge organization (CcmK in orange, CcmL in green at the vertices), according to the shell hexamer length (7.5 nm) and the edge length (72.2 nm) measured from TEM images. Middle, the structural model of one β-carboxysome module including one shell facet (orange), inner shell layer (grey) and a triangular pyramid Rubisco-organizing core (blue). Bottom, twenty of such β-carboxysome modules assemble to form the entire icosahedral β-carboxysome in Syn7942.

In contrast, EM images of the 40% fraction show the regular and polyhedral shape of intact β-carboxysomes from Syn7942 (Fig. 4A and Fig. S3†). These organelles exhibit an average diameter of 149.90 nm (Fig. 4B and Table 1), larger than the isolated α-carboxysomes from *Halothiobacillus neapolitanus*,^37^*Synechococcus* WH8102,^38^ and *Prochlorococcus marinus* MED4^32^ (Table S2†). Interestingly, the size of isolated β-carboxysomes is slightly smaller than that determined from previous thin-section TEM results.^19^,^41^ Nevertheless, unlike typical icosahedral viruses, β-carboxysomes vary in size, ranging from 100 to 200 nm (Fig. 4B), consistent with the observations from *in vivo* confocal fluorescence microscopy and TEM results.^19^,^22^,^28^,^36^,^40^ The structural heterogeneity implicitly indicates the inherent dynamics of β-carboxysome formation and biogenesis *in vivo*, which might be of physiological importance to the generation of new β-carboxysomes from pro-carboxysomes or pre-existing carboxysomes and the degradation of mature β-carboxysomes during cell growth and division.^28^,^35^ Moreover, two closely associated β-carboxysomes were occasionally seen (Fig. 4C and Fig. S3†). Despite the possibility of being artifacts in sample preparation, whether they are generated by potential interactions between neighboring carboxysomes or in the budding events^28^ remains unknown.

**Table 1:** Physical properties of the CcmK4:eGFP β-carboxysomes determined using EM and AFM in this study. The value column illustrates the means, the standard deviation errors and the units of the physical properties. The n column indicates the number of individual β-carboxysomes examined. The Detection Method column shows what techniques and methods were exploited to obtain the data. The structural dimensions of β-carboxysomes measured by EM was used to build the model illustrated in Fig. 4I and measure the mechanical properties (Fig. 6)

	Value	*n*	Detection method
Diameter	149.90 ± 13.78 nm	90	EM
Facet length	72.16 ± 7.51 nm	40	EM
Shell thickness	4.51 ± 0.22 nm	60	EM
RuBisCO packing periodicity	9.50 ± 0.70 nm	30	EM
Shell–core interval thickness	2.00 ± 0.24 nm	60	EM
Height	135.23 ± 23.02 nm	50	AFM
Spring constant *k*_CB_	20 ± 9 pN nm^–1^	25	AFM nanoindentation
Young's modulus *E*_H_	0.59 ± 0.34 MPa	25	AFM nanoindentation (Hertzian model)
Young's modulus *E*_S_	77.90 ± 23.89 MPa	25	AFM nanoindentation (Linear model)

Besides the overall shape of β-carboxysomes, EM images also provide detailed information about the shell architecture and internal organization of the β-carboxysome, which advance the model of β-carboxysome structure and assembly^13^,^23^ (Fig. 4D–F). It is evident that the intact β-carboxysome comprises an outer shell that incorporates paracrystalline arrays of Rubisco enzymes (Fig. 4F). The average length of shell facet edges (vertex to vertex) is 72.16 ± 7.51 nm (*n* = 240). The thickness of the outer shell is 4.51 ± 0.22 nm (*n* = 60) (Fig. 4G), in agreement with the thickness of a single CcmK2 protein.^17^ It demonstrates that the β-carboxysome shell is constructed with a single layer of shell proteins. Intriguingly, we also observed a 2.0 nm low-density interval between the shell layer and Rubisco arrays (2.00 ± 0.24 nm, *n* = 60). This “gap” may accommodate a layer of loosely-packed proteins attached to the inner surface of the shell, *e.g.* CcaA, CcmM and CcmN, which play key roles in linking the shell and Rubisco-organizing internal structure.^13^

In contrast to the relatively disordered and less densely packed α-carboxysome lumen,^32^,^37^,^38^,^42^ the β-carboxysome internal structure is highly defined with paracrystalline arrays of Rubisco, in line with EM results of the ruptured Syn7942 cells.^43^ Individual Rubisco molecules inside the β-carboxysome, notably those located in the outer layers of Rubisco arrays and adjacent to the shell, are clearly discriminated in the highly-ordered β-carboxysomal lumen. Approximately 9.5 nm Rubisco center-to-center distance was resolved (Fig. 4H). Given the 3.5 nm edge length of CcmK2 hexamer,^17^ the edge of a shell facet (72.16 ± 7.51 nm) is capable of accommodating 6 pairs of hexamers and 5 single hexamers between two CcmL pentamers at the vertexes (Fig. 4I, top). About 7 Rubisco proteins (∼10 nm each) can be located along each facet edge under the outer shell (Fig. 4I, middle). Such protein organization will result in a triangular pyramid β-carboxysome substructure, which contains one shell facet with a single hexamer thick, a layer of shell-associated proteins and a Rubisco-organizing triangular pyramid. Twenty of these β-carboxysome modules eventually construct the entire icosahedral β-carboxysome architecture (Fig. 4I, bottom). A Rubisco-organizing pyramid under a triangular shell facet is estimated to contain 84 Rubisco proteins; a total of 1680 Rubisco enzymes may be encapsulated in one β-carboxysome, roughly consistent with the previous estimation.^13^ Due to the paracrystalline packing, the Rubisco content of the β-carboxysome is 7-fold higher than that of the α-carboxysome,^32^,^38^ though absolute quantification is required to explore the exact abundance of protein modules in carboxysomes.

The different interior organizations of α- and β-carboxysomes could result in the distinction in their hierarchical assembly processes. The biogenesis of α-carboxysome was proposed to be initialized by the formation of outer shell^30^,^31^ or follow a simultaneous assembly pathway,^32^ whereas β-carboxysomes seem to assemble from the inside out.^28^,^29^ Characterization of partial β-carboxysomes in this work suggests the strong protein–protein interactions within the “outer shell–inner layer–Rubisco” structures. The shell proteins, shell-associated proteins and Rubisco enzymes could potentially co-assemble to form large carboxysome modules, which may serve as the assembly intermediates during β-carboxysome assembly, biogenesis or degradation. Concomitantly, our EM results of intact β-carboxysomes, showing more ordered Rubisco arrays at the outer surface of Rubisco arrays and less ordered Rubisco packing in the β-carboxysome lumen, likely implies the potential “outer shell–inner layer–Rubisco” interactions (Fig. 4F).

### Topography and physical properties of single β-carboxysomes

**Fig. 5:**
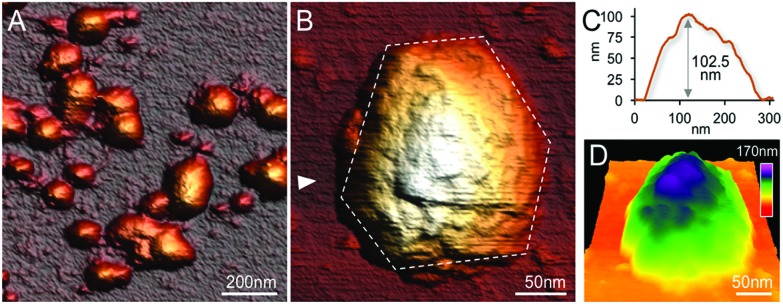
Native AFM topographs of intact β-carboxysomes from Syn7942. (A) An overview topograph of isolated intact β-carboxysomes in the 40% fraction captured by AFM in solution. (B) High-resolution AFM image of a single intact β-carboxysome, showing the morphological features of β-carboxysomes. Several surface protrusions can be distinguished. The polyhedral shape of the β-carboxysome is outlined by white dashed lines. (C) Height profile of the β-carboxysome, taken along the white arrow indicated in (B). (D) 3D representation of the native architecture of the same β-carboxysome from Syn7942.

High-resolution AFM imaging in solution has become a matured and powerful single-molecular tool in studying the structures of macromolecular complexes.^44^ By applying AFM imaging in solution, we characterized for the first time the topography and spatial protein organization of intact β-carboxysomes under near physiological conditions (Fig. 5). The identification and structural integrity of β-carboxysomes fused with eGFP were confirmed by simultaneous AFM-fluorescence imaging (Fig. S4†). AFM overview images illustrate the proper immobilization and distribution of individual β-carboxysomes on the substrate surface (Fig. 5A). High-resolution AFM images enable the direct characterization of the topography and dimension of individual β-carboxysomes (Fig. 5B, C and Table 1). The average height of β-carboxysomes is 135.23 ± 23.02 nm (*n* = 50), consistent with TEM results (Fig. 5C). Substructures in the β-carboxysome surface were readily discerned at this resolution, which represents the molecular organization of the β-carboxysome shell (Fig. 5B–D). The facet boundaries could be occasionally observed in single carboxysomes (Fig. S5†). Individual shell protein structures on intact β-carboxysomes could not be distinctly discerned at this resolution, compared to partial β-carboxysome structures that are better supported by the AFM substrate (Fig. 3). These observations suggest the softness and flexible conformation of β-carboxysomes. The aggregation of two β-carboxysomes was also visualized in AFM (Fig. S5†), in line with our EM observation (Fig. 4C and Fig. S3†).

**Fig. 6:**
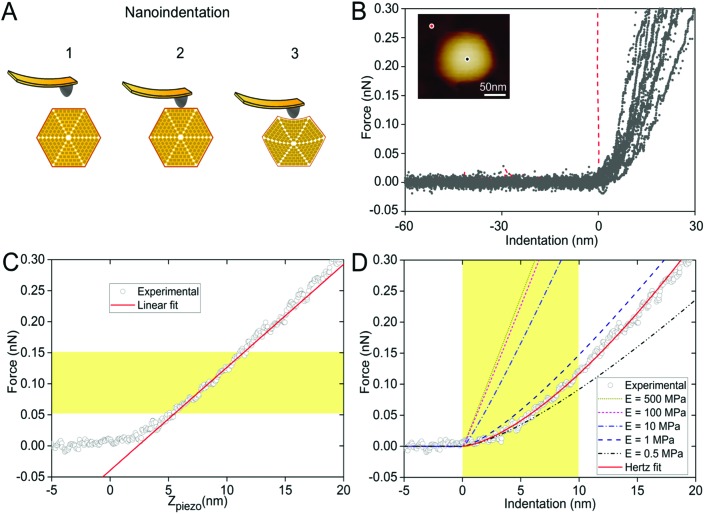
Mechanical characterization of intact β-carboxysomes using AFM nanoindentation. (A) Schematic of an AFM nanoindentation experiment, including AFM tip engagement (1), tip-carboxysome contact (2) and indentation (3) with increasing force. (B) Force-indentation curves of individual β-carboxysomes. The red curve is the reference curve on the mica substrate. Inset, AFM image of a single β-carboxysome during AFM nanoindentation. The black dot represents the indentation position on the carboxysome, whereas the red dot represents the indentation position on mica surface. (C) A typical force-displacement curve of a single β-carboxysome. The red line is the fitting using the linear model based on the 0.05–0.15 nN region of the force curve. (D) A typical experimental (circle) force-indentation curve of a single β-carboxysome and simulated force-indentation curves (colored dash lines) using a Hertz contact model in a sample with Young's modulus ranging from 0.5 to 500 MPa. The red curve is the fitting using the Hertzian model based on the 0–10 nm region of the force curve. Young's modulus of β-carboxysomes (*E*_H_) is 0.59 ± 0.34 MPa (*n* = 25), sitting between the predicted Young's moduli of 0.5 and 1 MPa.

In the crowded and dynamic cellular environment, the physical properties of bacterial organelles are essential for their stability, functionality and regulatory responses.^45^ Using AFM-based nanoindentation that has been exploited in studying viral capsid mechanics,^46^ we determined the spring constant and Young's modulus of β-carboxysomes to unveil, for the first time, the mechanical properties of carboxysomes at near physiological conditions (Fig. 6). A relatively low force (100 pN) was applied for AFM imaging and 1 nN force was applied for AFM nanoindentation on targeted β-carboxysomes. Fig. 6A shows schematically a typical nanoindentation event performed on an intact β-carboxysome. After locating the β-carboxysome by AFM imaging, the AFM tip was positioned over the center of the β-carboxysome (Fig. 6B, inset) and pushed towards the organelle (stage 1). There is zero force with *z*-displacement until the tip and carboxysome contact (stage 2). As the tip pushes down, there is an increase in the force, resulting in the deformation of the β-carboxysome structure (stage 3). Fig. 6B exhibits a collection of force-indentation curves of β-carboxysomes. Within the range of 0–300 pN, the indentation on β-carboxysome is up to 20 nm, which represents about 10% of the particle height, according to the previous study.^47^ No typical rupture/breaking events, as seen in viruses, were observed in β-carboxysomes above 300 pN (data not shown). A typical force-displacement curve, as depicted in Fig. 6C, illustrates an initial nonlinear response, followed by a relatively linear deformation of the β-carboxysome. The slope of the linear-like regime of the force-indentation curve is the spring constant *k* of β-carboxysomes (∼20 pN nm^–1^, *n* = 25, eqn (1), Fig. 6C and Fig. S6†), which represents the stiffness of β-carboxysomes (Fig. 6C). The spring constant of β-carboxysomes is lower than those typical for viruses (*k*: 40–1250 pN nm^–1^),^48^–^54^ revealing that the β-carboxysome is softer than the viruses with protein-based shells.

The thin-shell model has been widely used to determine Young's moduli of viruses, which have a linear elastic response to the indentation.^55^,^56^ In contrast, the force–distance curves of β-carboxysomes present evidently the nonlinear nature (Fig. 6C) indicating the flexibility of the carboxysomal structures. Thus, the overall force-indentation curve is fitted to the Hertzian model^57^ in the 0–10 nm region of the force curves (Fig. 6D and eqn (3)), to obtain Young's modulus of β-carboxysomes (*E*_H_ = 0.59 ± 0.34 MPa, *n* = 25, Table 1 and Fig. S6†). Fig. 6D also shows the force-indentation curves obtained from experimental data and simulations with Young's modulus ranging from 0.5 to 500 MPa. The experimental curve (0.59 ± 0.34 MPa) sits exactly between the simulated curves with Young's moduli of 0.5 and 1 MPa. It exhibits notably lower Young's modulus than the bacterial nanocompartment encapsulin (*E*_H_ for encapsulin: 30–60 MPa).^58^

As β-carboxysomes structurally resemble the virus capsids, we performed the comparison of the physical mechanics of carboxysomes and viruses. We calculated Young's modulus *E*_S_ of β-carboxysomes (77.90 ± 23.89 MPa, *n* = 25, Fig. S6†) using the thin-shell model (eqn (2)) in the 50–150 pN region of the force curves, based on the spring constant *k*, shell thickness (*h* = ∼4.5 nm) and the size of β-carboxysomes (*R* = ∼75 nm) which were determined from our AFM and TEM imaging (see Table 1). The estimated *E*_S_ is much lower than those of viruses (140 MPa–1.8 GPa) and encapsulin (1.2–2.0 GPa).^55^,^58^ It is worthy to note that the exact thickness of the β-carboxysome “shell” remains to be determined to obtain accurate Young's modulus of β-carboxysomes, given the presence of the shell inner layer that is composed of shell-associated proteins observed in the TEM images (Fig. 4F).

We further compared the nanomechanical features of β-carboxysomes and *Salmonella typhimurium* bacteriophage P22 (Fig. S7†). The physical mechanics of P22 has been characterized previously.^53^,^59^,^60^ To confirm the reliability of our mechanical measurement, we applied the same procedure of AFM imaging and nanoindentation to P22. The height of P22 particles is 65.1 ± 5.9 nm (*n* = 20) and the spring constant of P22 is 192.38 ± 63.77 pN nm^–1^ (*n* = 8), comparable to previously published results.^53^,^60^ Young's moduli of P22 fitted to the linear model and the Hertzian model are 101.04 ± 32.29 MPa and 11.06 ± 8.77 MPa, respectively (*n* = 8). They are both higher than those of β-carboxysomes, suggesting that the β-carboxysome exhibits the softer mechanics than P22 (Fig. S7†).

Carboxysomes architecturally resemble icosahedral virus capsids. However, there is no evidence for sequence or structural similarity of carboxysome shell proteins to known viral capsid proteins.^4^,^61^,^62^ An open question is whether carboxysomes have the same rigidity as viruses. Here, we show that the particle stiffness and intrinsic rigidity, represented by the spring constant and Young's modulus of β-carboxysomes, are both weaker in contrast to those of the human Herpes simplex virus type 1 (HSV-1) capsid and adenovirus, which have comparable dimensions.^47^,^52^,^63^ Interestingly, β-carboxysomes exhibit similar stiffness with the influenza virus which contains a lipid envelope,^48^ whereas they are much softer compared to the icosahedral encapsulin.^58^ Nevertheless, our results reveal the mechanical softness and flexibility of β-carboxysomes in contrast to rigid virus capsids, likely ascribed to the specific assembly of multiple protein homologs in the complex carboxysomal shell architecture. Such unique mechanical signature of β-carboxysomes might be essential to the functional plasticity of the metabolic machinery in response to environmental changes, and facilitate the metabolite passage, turnover of building blocks, recognition and regulation by other cellular components. The soft and flexible architecture could make it difficult to easily define the edges of β-carboxysomes and individual shell proteins by AFM imaging in solution even with gentle scanning force (100 pN), though β-carboxysomes display regular polyhedral shape in TEM images. Applying more gentle scanning and sample fixation might be of help to obtain higher-resolution images.

Apart from the shell composition, a striking difference between the carboxysome and viruses is the internal organization. The viral genome is enclosed within the viral capsid, whereas the carboxysome contains densely arranged enzymes inside the shell. It is unclear how the packing of Rubisco enzymes and protein–protein interactions within the β-carboxysome have impacts on the overall architecture and mechanical properties of the shell and intact carboxysome. It was shown that “empty” β-carboxysome shells in the absence of Rubisco enzymes are only 20–30 nm in diameter.^64^ Further study is required to uncover what determines the assembly and intrinsic mechanics of β-carboxysomes.

## Conclusions

Carboxysomes are the key metabolic modules for carbon fixation in cyanobacteria and show great promise for synthetic engineering to improve the catalytic efficiency of enzymes in non-native hosts. In this work, we conducted the isolation of functional β-carboxysomes from the cyanobacterium Syn7942 and the direct visualization of the native organization, topography and intrinsic mechanics of β-carboxysomes using TEM, AFM and proteomics. We find that the intact β-carboxysome poses three distinct structural domains, a single-layered icosahedral shell, an inner layer and paracrystalline arrays of interior Rubisco. We also characterized partial β-carboxysome structures that consist of shell facets, shell-associated proteins as well as Rubisco enzymes, probably serving as the assembly intermediates of β-carboxysomes. In addition, we applied AFM to directly characterize the native protein organization of shell facets and the topography and intrinsic mechanics of native β-carboxysomes for the first time. Our results illustrate the soft nanomechanical properties of β-carboxysomes compared to icosahedral viruses, likely revealing the unique spatial organization of carboxysomal building blocks. The study provides new insights into the assembly, organization and physical nature of functional β-carboxysomes, which can be extended to α-carboxysomes and other BMCs. Comprehensive understanding of the carboxysome structure and mechanics will underpin the design and engineering of functional synthetic carboxysomes, to enhance photosynthesis and develop new bio-nanoreactors and protein scaffolds using BMC proteins as nanoscale materials. It offers a powerful approach for assaying the functional organizations and material mechanics of natural and engineered biological systems.

## Materials and methods

### Bacterial strain and growth conditions

The CcmK4:eGFP strain of Syn7942 was generated previously.^36^ Syn7942 cultures were maintained in constant illumination in BG-11 medium and grown in a 5-liter fermenter (BioFlo 115, New Brunswick Scientific, USA) at 30 °C under 100 μE m^–2^ s^–1^ with constant agitation and bubbling with air. The growth of cultures was tracked using OD 750 nm measurements by spectrophotometer (Jenway 6300 Spectrophotometer, Jenway, UK).

### β-Carboxysome isolation

Cells were harvested at OD = ∼3.5 before reaching stationary phase. All subsequent steps were carried out at 4 °C and the resulting samples were stored at 4 °C. The cell pellet was resuspended and the presence of 2% cell lytic B (Sigma Aldrich, US), 1% protease inhibitor cocktails (Thermo-Fisher, UK) and 10 mg ml^–1^ lysozyme (Sigma Aldrich, US), for 1 hour prior to cell breakage by sonication. Cell lysate was then treated with 3% Trion X-100 (Sigma Aldrich, US) for 1 hour. Cell debris was removed by centrifugation, followed by a centrifugation at 50 000*g* to enrich β-carboxysomes and discard some cellular components in the supernatant. The generated pellet was resuspended in TE buffer and was incubated in the presence of 1% *n*-doceyl β-maltoside (Sigma Aldrich, US), followed by centrifugation using a step sucrose gradient. Each sucrose fraction was characterized by fluorescence microscopy, SDS-PAGE and Rubisco assay to determine the presence and activities of β-carboxysome components.

### SDS-PAGE and immunoblot analysis

SDS-PAGE and immunoblot analysis were carried out as previously described.^36^

### Rubisco assay

Isolated β-carboxysome samples were diluted to 1 mg ml^–1^ protein concentration by Bradford Assay using Rubisco assay buffer (100 mM EPPS, pH 8.0; 20 mM MgCl_2_). These samples were then added into scintillation vials containing NaH^14^CO_3_ final concentration 25 mM and incubated at 30 °C for 2 min before the addition of d-ribulose 1,5-bisphosphate sodium salt hydrate (RuBP, Sigma Aldrich, US) final concentration 1 mM. The reaction ensued for 5 min before being terminated by adding 2 : 1 by volume 10% formic acid. Samples were dried for at least 30 min at 95 °C to remove unfixed ^14^C before re-suspending the fixed ^14^C pellets with ultra-pure water and adding 2 ml of scintillation cocktail (Ultima Gold XR, PerkinElmer, US). Radioactivity measurements were then taken using a scintillation counter (Tri-Carb, PerkinElmer, US). Raw readings were used to calculate the amount of fixed ^14^C, and then converted to the total carbon fixation rates. Results are presented as mean ± standard deviation (SD).

### Proteomic analysis

The β-carboxysome sample from the 40% sucrose fraction was washed with PBS buffer. Rapigest was added to a final concentration of 0.05% (w/v) into the sample for 10 min incubation at 80 °C. The sample was then reduced with dithiothreitol (3 mM, final concentration) for 10 min at 60 °C, alkylated with iodoacetamide (9 mM, final concentration) for 30 min at room temperature in the dark, followed by digestion with trypsin at 37 °C overnight. Digestion was terminated with 1 μL of trifluoroacetic acid (TFA). Data-dependent LC-MS/MS analysis was conducted on a QExactive quadrupole-Orbitrap mass spectrometer coupled to a Dionex Ultimate 3000 RSLC nano-liquid chromatograph (Hemel Hempstead, UK). 2 μL sample digest was loaded onto a trapping column (Acclaim PepMap 100 C18, 75 μm × 2 cm, 3 μm packing material, 100 Å) in 0.1% TFA, 2% acetonitrile H_2_O, and set in line with the analytical column (EASY-Spray PepMap RSLC C18, 75 μm × 50 cm, 2 μm packing material, 100 Å). Peptides were eluted using a linear gradient of 96.2% buffer A (0.1% formic acid) : 3.8% buffer B (0.1% formic acid in water : acetonitrile 80 : 20, v/v) to 50% buffer A : 50% buffer B over 30 min at 300 nL min^–1^. The mass spectrometry analysis was operated in DDA mode with survey scans between *m*/*z* 300–2000 acquired at a mass resolution of 70 000 (FWHM) at *m*/*z* 200. The maximum injection time was 250 ms, and the automatic gain control was set to 1e^6^. Fragmentation of the peptides was performed by higher-energy collisional dissociation using a normalized collision energy of 30%. Dynamic exclusion of *m*/*z* values to prevent repeated fragmentation of the same peptide was used with an exclusion time of 20 seconds.

The raw data file was imported into Progenesis QI for Proteomics (Version 3.0 Nonlinear Dynamics, Newcastle upon Tyne UK, a Waters Company). Peak picking parameters were applied with sensitivity set to maximum and features with charges of 2^+^ to 7^+^ were retained. A Mascot Generic File, created by Progenesis, was searched against the Syn7942 database from UniProt (2657 proteins) with the sequence of yeast enolase (UniProt: P00924) added. Trypsin was specified as the protease with one missed cleavage allowed and with fixed carbamidomethyl modification for cysteine and variable oxidation modification for methionine. A precursor mass tolerance of 10 ppm and a fragment ion mass tolerance of 0.01 Da were applied. The results were then filtered to obtain a peptide false discovery rate of 1%. Protein quantification was calculated using Hi3 methodology using yeast enolase (50 fmol μL^–1^) as a standard protein.

### TEM imaging and image analysis

The structures of isolated β-carboxysomes were characterized using negative staining TEM as described previously.^65^,^66^ Samples were stained with 3% uranyl acetate. Images were recorded using a FEI Tecnai G2 Spirit BioTWIN FEI transmission electron microscope. Image analysis was carried out using ImageJ software (NIH Image). Results are presented as mean ± SD.

### AFM imaging, confocal-AFM imaging and nanoindentation

All AFM experiments were carried out in solution to ensure the structural and functional integrity of β-carboxysomes. Purified β-carboxysomes were adsorbed onto freshly cleaved mica surface at room temperature in TN buffer (10 mM Tris, 5 mM NiCl_2_, pH 8.0) for 1 hour, and then washed and imaged with TN buffer. AFM imaging was operated at room temperature on a MultiMode 8 AFM with NanoScope V controller (Bruker, Santa Barbara, US) in peak force tapping mode in liquid. AFM tips with the spring constant of 0.4 N m^–1^ (Scanassyst air HR, Bruker, Santa Barbara, US) were used for high-resolution imaging and the tip spring constant was routinely calibrated. The average imaging force was ∼100 pN.^67^ Confocal-AFM images were captured using a NanoWizard 3 AFM (JPK) integrated with a Zeiss LSM880 confocal microscope. Samples were adsorbed on glass slides in adsorption buffer (10 mM Tris-HCl, 150 mM KCl, 25 mM MgCl_2_, pH 7.5) for 10 min, and then washed with imaging buffer (10 mM Tris-HCl, 150 mM KCl, pH 7.5). Confocal images were captured using a 40× objective with 488 nm excitation. Particles with high-intensity GFP signal were imaged by AFM in Quantitative Imaging (QI) mode. The scanning force is ∼100 pN. Image analysis was performed using NanoScope Analysis (Bruker), JPK SPM Data Processing (JPK), WSxM^68^ and Igor Pro (WaveMetrics).

Force spectroscopy measurements were performed using V-shaped, silicon nitride cantilevers (DNP, Bruker, USA) with a tip radius *R* = 20 nm, nominal spring constant *k* = 0.35 N m^–1^ (for Bruker AFM) and *k* = 0.06 N m^–1^ (for JPK AFM). Typically, 3 to 5 force curves were acquired at different positions of the central region of the carboxysome, up to a maximum applied force of 1 nN, at indentation speed of 200–300 nm s^–1^. Force–distance curves with the indentation of around 10 nm were acquired in the top region of the carboxysomes to determine the elastic properties of carboxysomes.

Assuming that β-carboxysomes could have a mechanical behaviour similar to that of viruses, we used three typical models to determine its mechanical properties. The first one is the linear model, widely used to study virus rigidity, where the cantilever and the particle are considered as two springs in series.^69^,^70^ The spring constant of β-carboxysomes *k*_CB_ was calculated using:1
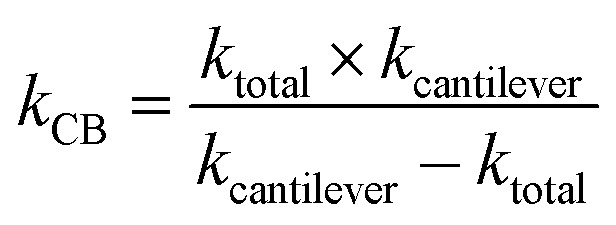
where *k*_cantilever_ was the pre-calibrated spring constant of the cantilever and *k*_total_ was the slope measured in the range of interest of the force–distance curve recorded on top of the β-carboxysome.

Stiffness is a property of the object and depends on its material, but also on its dimensions and geometry.^70^ Young's modulus provides a measurement of the intrinsic elasticity of the material. In the case of viruses, is very common the use of the thin-shell theory to estimate *E*_S_.^55^,^70^ Young's modulus can be estimated using the following equation:^71^2
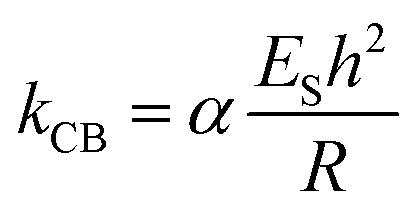
where *α* is the geometry-dependent proportionality factor (here we consider *α* = 1), which was reported to be a reasonable value for various virus capsids,^69^*k*_CB_ is the spring constant of β-carboxysomes, estimated using the lineal model (eqn (1)), *h* and *R* are the shell thickness and the particle's radius measured by TEM (4.5 nm and 75 nm, respectively)

The third model used to estimate the mechanical properties of β-carboxysomes is the Hertzian model.^57^ This model is implemented in the commercial software of the Bruker and the JPK systems. If the sample is softer than the tip, Young's modulus *E*_H_ can be obtained using:3
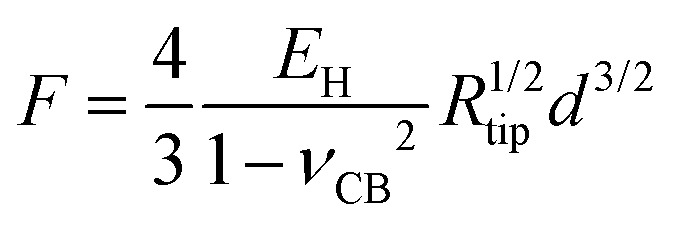
where *F* is the measured force, *R*_tip_ is the tip radius (for DNP cantilevers, *R* = 20 nm), and *ν*_CB_ is the Poisson coefficient of β-carboxysomes (here we consider *ν*_CB_ = 0.5, for soft biological samples^72^) and *d* is the indentation depth, determined from the displacement *z*_p_ of the piezo-scanner, the initial contact distance *z*_0_, and the deflection given by a hard wall *F*/*k*_cantilever_:4
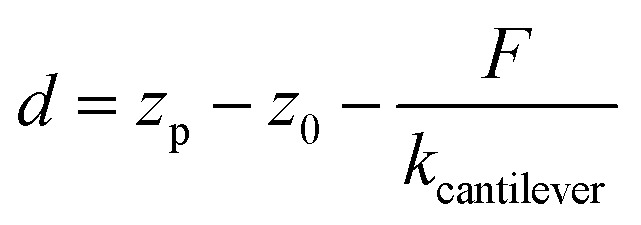



Simulations of force-indentation curves were carried out using the Force Distance Curves tool in the Virtual Environment for Dynamic AFM (VEDA) software,^73^ assuming a Hertz contact regime. The cantilever was assumed to have a tip radius *R* = 20 nm, spring constant *k* = 100 pN nm^–1^, Young's modulus *E*_t_ = 130 GPa and Poisson coefficient *μ*_s_ = 0.3. For carboxysomes, a Poisson coefficient *μ*_s_ = 0.5 was used and Young's modulus was in the range from 0.5 to 500 MPa.

### P22 bacteriophage isolation, AFM imaging and nanoindentation

Wild type P22 bacteriophages were propagated in a *Salmonella typhimurium* strain, D23580 Δ*Φ*, which has been cured for all functional prophages.^74^ When the culture reached approximately OD = 0.2, 10 μl of wild type P22 single-plaque suspension was added and the cells were cultured at 37 °C for 12 hours for the replication of viruses. A pure suspension of P22 viruses was purified by filtration through a 0.22 μm filter, with a concentration of 10^11^ virus particles per ml. Preparation of P22 bacteriophages on mica, AFM imaging and nanoindentation were carried out using the same parameters as for β-carboxysomes for direct comparison. Young's modulus of P22 was calculated in the same manner as that of the β-carboxysome. *E*_SP22_ was obtained by fitting the linear model (eqn (2)), where the spring constant *K*_P22_ was obtained from our experimental data, *α* is 1, *R* is 30 nm and *h* is 7.5 nm.^60^*E*_HP22_ was obtained by fitting the Hertzian model (eqn (3)).

## Supplementary Material

Supplementary information
