# Supplementary material for: Direct characterization of the native structure and mechanics of cyanobacterial carboxysomes
Source: Nanoscale. 2017 Jun 8;9(30):10662–73. doi: 10.1039/c7nr02524f (PMC5708340; doi:10.1039/c7nr02524f)
Supplement: Supplementary file 1 [file NR-009-C7NR02524F-s001.pdf]

Electronic Supplementary Material (ESI) for Nanoscale.
This journal is © The Royal Society of Chemistry 2017

Supporting Information

Direct characterization of the native structure and mechanics of cyanobacterial carboxysomes

Matthew Faulkner,[a] Jorge Rodriguez-Ramos,[a] Gregory F. Dykes,[a] Sian V. Owen,[a] Selene Casella,[a] Deborah M. Simpson,[a] Robert J. Beynon[a] and Lu-Ning Liu[a,*]

[a]*Institute of Integrative Biology, University of Liverpool, Liverpool L69 7ZB, United Kingdom. E-mail: luning.liu@liverpool.ac.uk.*

Table S1 Proteomic results of isolated β-carboxysomes from Syn7942. The column of Normalized amount displays the amount of each of the carboxysomal proteins detected in isolated β-carboxysomes using mass spectroscopy, normalized against the amount of the least abundant protein CcmL.

Protein	Normalized amount (fmol)
RbcL	4530.8 ± 432.8
RbcS	1744.5 ± 765.1
CcmM	1567.1 ± 412.8
CcmK2	116.6 ± 22.0
CcaA	81.8 ± 12.6
CcmK4	18.7 ± 0.4
CcmL	1

Table S2 Comparison of the dimensions of isolated carboxysomes from different species using TEM.

Type	Species	Diameter (nm)	Range (nm)	Reference
α-carboxysome	*Halothiobacillus neapolitanus*	117.3 ± 6.9	97 – 132	[1]
α-carboxysome	*Halothiobacillus neapolitanus*	100	88 – 108	[2]
α-carboxysome	*Halothiobacillus neapolitanus*	134 ± 8	116 – 169	[3]
α-carboxysome	*Synechococcus* WH8102	123 ± 5	114 – 137	[4]
α-carboxysome	*Prochlorococcus marinus* MED4	90	70 – 100	[5]
β-carboxysome	*Synechococcus elongatus* PCC7942	149.90 ± 13.78	100 – 200	This study

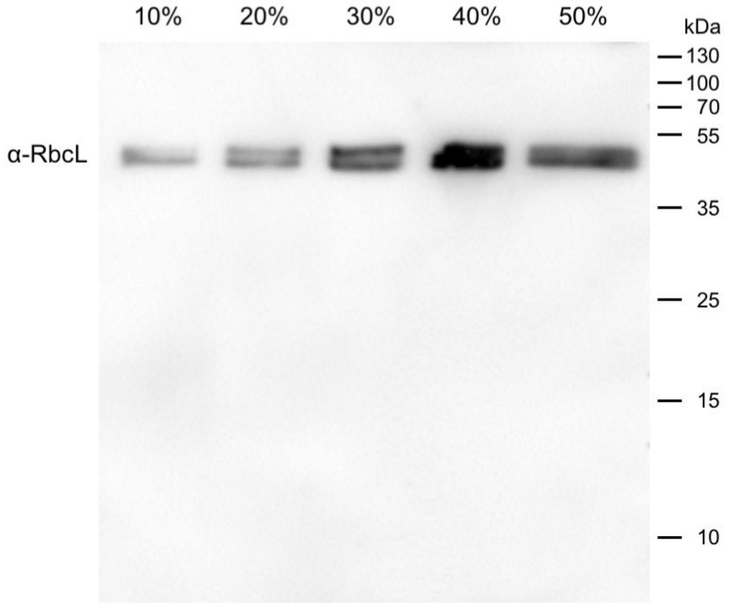

Fig. S1 Immunoblotting analysis of different β-carboxysome fractions using anti-RbcL antibody. Immunoblotting assays were carried out on the SDS-PAGE gel shown in Fig. 1C. RbcL (~50 kDa) was detected in all sucrose fractions and was most abundant in the 40 % sucrose fraction.

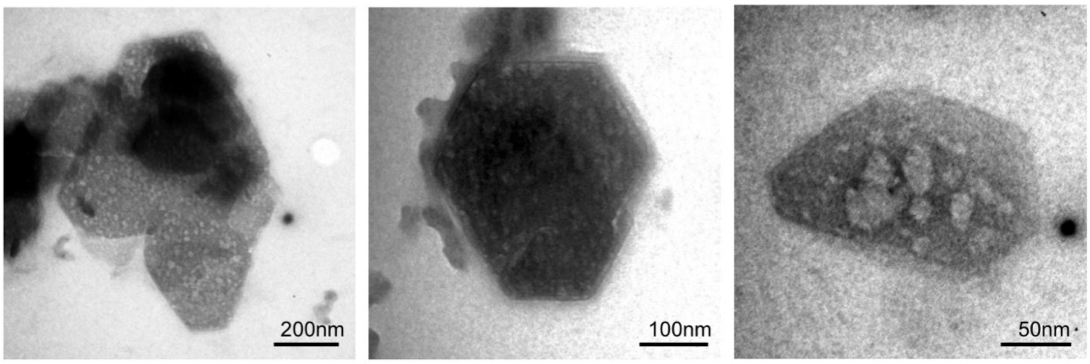

Fig. S2 TEM images of partial β-carboxysome fragments in the 20 and 30% sucrose fractions.

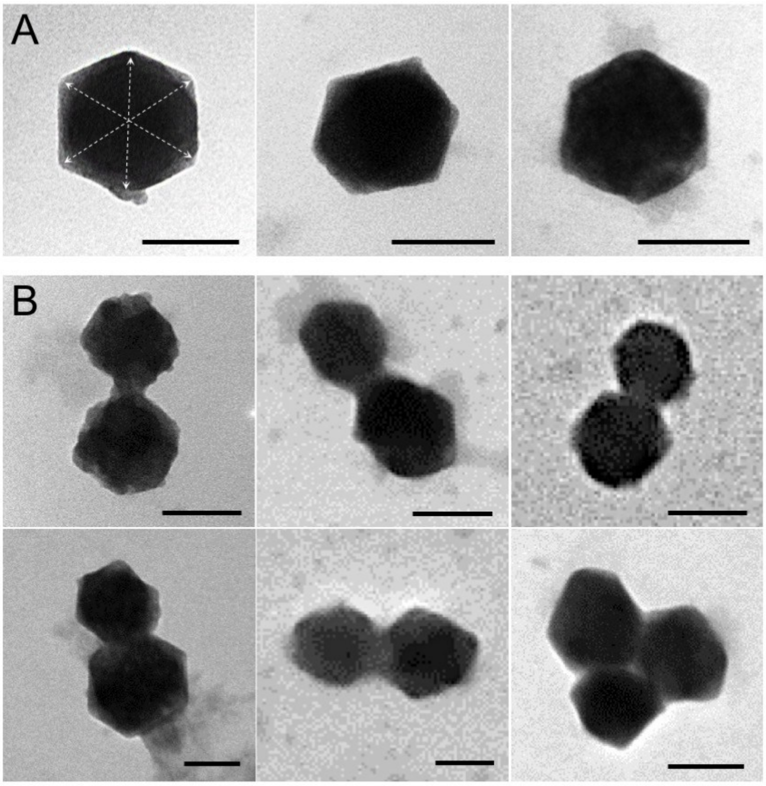

Fig. S3 TEM images of intact β-carboxysomes in the 40% sucrose fraction. (A) TEM images of individual intact β-carboxysomes. The dashed arrows represent the vertex-to-vertex measurements for determining the β-carboxysome diameter as described in Fig. 4B. (B) TEM images of β-carboxysome aggregations (Fig. 4C). Scale bar: 100 nm.

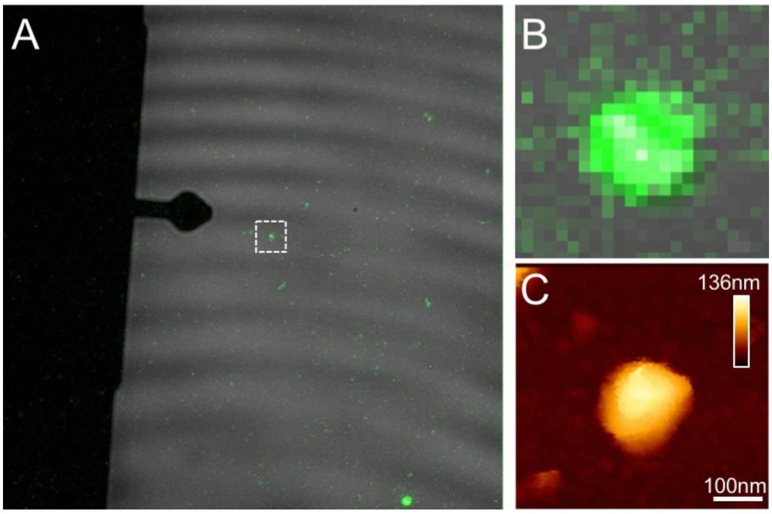

Fig. S4 Combined confocal and AFM imaging of β-carboxysomes fused with GFP. (A) A merged image of the transmitted and GFP channels captured using a hybrid JPK AFM-Zeiss 880 confocal microscope. The white dashed square represents a 10 × 10 µm field of view of AFM after the engage. (B) Fluorescence image of a single β-carboxysome in the view highlighted by the white square in panel A. (C) AFM topograph of the same β-carboxysome captured simultaneously with the fluorescence image (B). The combination of AFM-confocal fluorescence imaging ensures the identification of β-carboxysomes on AFM substrate.

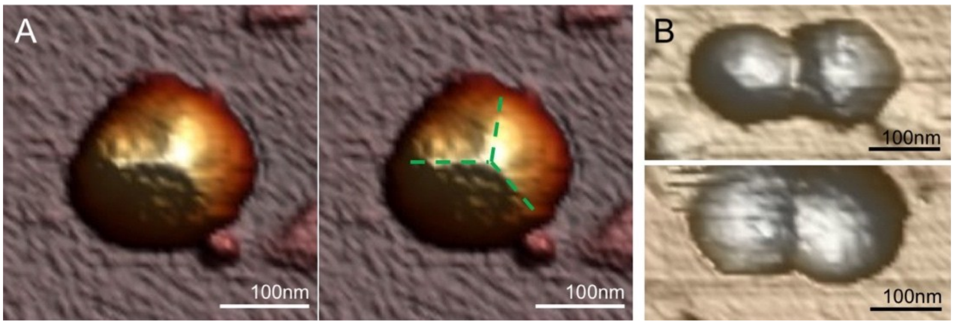

Fig. S5 AFM images of intact β-carboxysomes. (A) AFM topograph of single β-carboxysome with a vertex and three facet boundaries resolved, indicated by the green dashed lines. (B) AFM topographs of aggregated β-carboxysomes, reminiscent of EM results (Fig. 4C, Fig. S3B).

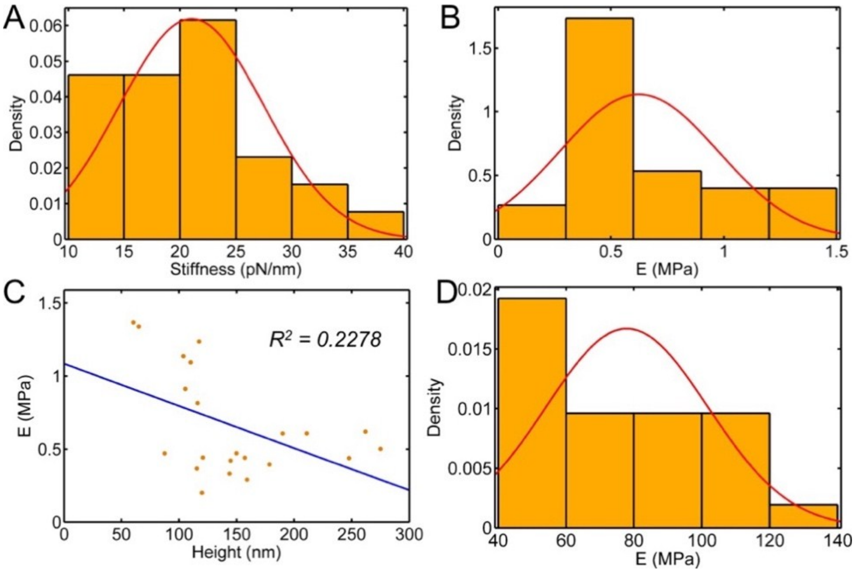

Fig. S6 Statistical analysis of the nanomechanical properties of β-carboxysomes. (A) Histogram of the β-carboxysome stiffness (k_{CB}, n = 25, Equation 1). (B) Histogram of Young's moduli of β-carboxysomes (E_H, n = 20) using the Hertzian model (Equation 3). (C) There is no correlation between Young's moduli and the carboxysome diameter (y = -0.0039x + 1.1, R^2 = 0.2278). (D) Histogram of Young's moduli of β-carboxysomes (E_S, n = 25) using the thin shell model (Equation 2).

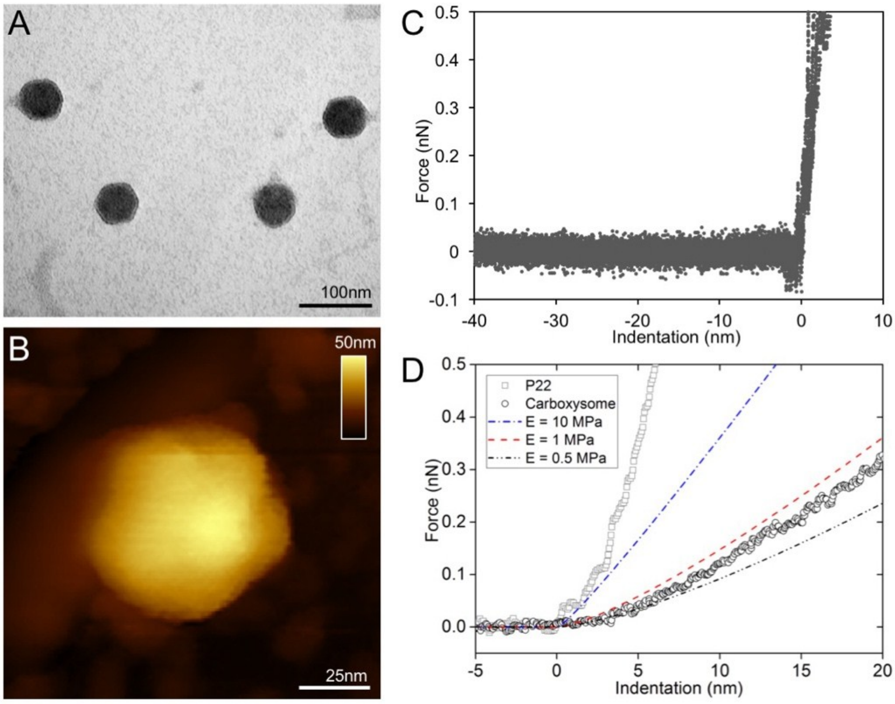

Fig. S7 Characterization of P22 particles. (A) TEM images of isolated P22 bacteriophage. (B) AFM topograph of a single P22 bacteriophage. The average height is 65.1 ± 5.9 nm (*n* = 20), in good agreement with previous AFM data.[6] (C) Force-indentation curves of individual P22 particles. (D) The force-indentation curves of a single β-carboxysome (circle), a single P22 particle (square) and simulated force-indentation curves (colored dash lines) using a Hertz contact model in a sample with Young's moduli of 0.5, 1 and 10 MPa. The height of P22 particles is 65.1 ± 5.9 nm (*n* = 20) and the spring constant of P22 is approximately 192.38 ± 63.77 pN/nm (*n* = 8). Young's moduli of P22 fitted to the linear model and the Hertzian model are 101.04 ± 32.29 MPa and 11.06 ± 8.77 respectively (*n* = 8). Young's modulus of β-carboxysomes obtained using the Hertzian model (E_H = 0.59 ± 0.34 MPa, *n* = 20) is significantly lower than those of P22, demonstrating the mechanical softness of β-carboxysome structures compared with P22.

Supporting References

1. Y. A. Holthuijzen, J. F. L. van Breemen, W. N. Konings and E. F. J. van Bruggen, *Archives of Microbiology*, 1986, **144**, 258-262.
2. M. F. Schmid, A. M. Paredes, H. A. Khant, F. Soyer, H. C. Aldrich, W. Chiu and J. M. Shively, *J Mol Biol*, 2006, **364**, 526-535.
3. C. V. Iancu, D. M. Morris, Z. Dou, S. Heinhorst, G. C. Cannon and G. J. Jensen, *J Mol Biol*, 2010, **396**, 105-117.
4. C. V. Iancu, H. J. Ding, D. M. Morris, D. P. Dias, A. D. Gonzales, A. Martino and G. J. Jensen, *J Mol Biol*, 2007, **372**, 764-773.
5. E. W. Roberts, F. Cai, C. A. Kerfeld, G. C. Cannon and S. Heinhorst, *J Bacteriol*, 2012, **194**, 787-795.
6. A. Llauro, D. Luque, E. Edwards, B. L. Trus, J. Avera, D. Reguera, T. Douglas, P. J. Pablo and J. R. Caston, *Nanoscale*, 2016, **8**, 9328-9336.
